# Impact of Tennis‐Specific Hitting and Running Loads on Respiration Patterns Compared to Treadmill Running With Similar Oxygen Uptake

**DOI:** 10.1002/ejsc.70088

**Published:** 2025-11-27

**Authors:** Katharina Raasch, Antonia Edel, Alexander Ferrauti

**Affiliations:** ^1^ Department for Training and Exercise Science Faculty of Sports Science Ruhr University Bochum Bochum Germany

**Keywords:** breathing plateaus, press breathing, racket sports, stroke velocities, tidal volume

## Abstract

This study aimed to compare respiratory patterns and gas exchange during tennis and treadmill running at similar oxygen uptake. On three experimental days, 15 elite players (7 female, 8 male) completed a standardized tennis protocol (TP), a treadmill‐based incremental running test (RT), and a metabolically matched running protocol (RP). TP (Day 1) included low or high running loads (RL, RH) and low or high stroke velocities (SL, SH) which were combined in four incremental ball‐machine stages (TP_1_:RL + SL; TP_2_:RL + SH; TP_3_:RH + SL; TP_4_:RH + SH). RT (Day 2) determined running speeds matching the oxygen uptake of each TP stage. RP (Day 3) replicated TP's mean oxygen consumption and stage duration through treadmill running. Metabolic and respiratory responses (portable spirometry) and external loads (stroke velocity) were compared between TP and RP. In tennis, mean oxygen uptake (*p* < 0.001), energy expenditure (*p* < 0.001), and respiratory exchange ratio (*p* < 0.001) as well as respiratory responses increased significantly between Stage 1–4. No differences in metabolic responses as well as in breathing frequency (bf), ventilation ($\dot{V}$’E), inspiration (tI), and expiration time (tE) were observed between TP and RP. In contrast, the number of breathing plateaus per stage (NP) was significantly higher in TP compared to RP during TP_2_ (5.9 ± 3.8 vs. 0.4 ± 0.5 times, *p* < 0.001), TP_3_ (2.6 ± 2.7 vs. 0.3 ± 0.6 times, *p* = 0.04), and TP_4_ (4.6 ± 4.1 vs. 0.3 ± 0.6 times, *p* < 0.001). Breathing plateaus appear characteristic for tennis when hitting hard without affecting metabolism. Exploring individual breathing patterns can be recommended in practice.

## Highlights

- Respiration patterns differ markedly between tennis and treadmill running at similar mean oxygen uptake.
- Breathing plateaus occur exclusively in tennis and are utilized by most players without increasing post‐exercise oxygen consumption.
- Different breathing strategies during on‐court drills may help players and coaches enhance individual performance.

## 1 Introduction

Physical activity comes along with an increase of energy expenditure and oxygen uptake, which leads to a stimulation of breathing activity and an overall increase of the respiratory volume (Forster et al. 2012; Harbour et al. 2022). In many continuous sports, breathing is often synchronized with movement through a phenomenon called locomotor‐respiratory coupling (LRC), which enhances breathing efficiency (Vickery 2008; Harbour et al. 2022, 2023; van Rheden et al. 2023). LRC can manifest both consciously and unconsciously, and it appears to be more prevalent among experienced athletes (Harbour et al. 2022). For instance, competitive freedivers utilize hyperinflation before a dive to maximize lung volume (Fitz‐Clarke 2018). Swimmers implement phased breathing, inhaling during specific phases of the swim stroke when their face is above water (Harbour et al. 2023). In cross‐country skiing, a close coupling between cycle rate and breathing rate is observed during the double pole technique (Björklund et al. 2015). As training intensity increases, rowers adjust their breathing/stroke ratio from 1:1 to 2:1 (Fabre et al. 2007). In running, breathing frequency is coordinated with step cadence to control metabolic load while reducing from four to two steps per inhalation and exhalation with increasing running velocity (Harbour et al. 2023; van Rheden et al. 2023). Additionally, an unconscious coupling of breathing rate to cadence has been demonstrated in triathletes (Bonsignore et al. 1998; Vickery 2008).

Although the coupling of breathing frequency and movement is well‐established in continuous sports, racket sports present unique challenges due to their semi‐continuous nature, characterized by frequent changes in footwork, stroke production, and court coverage (Fernández et al. 2009; Fernandez‐Fernandez et al. 2010; Edel et al. 2019; Cádiz Gallardo et al. 2023). Given their intermittent demands, a distinctly different breathing pattern can be expected compared to continuous sports. It is assumed that players in racket sports intuitively select an appropriate breathing technique in relation to intensity and skill production. Therefore, it is not surprising that tennis players seem to synchronize breathing with hitting. Many professional players such as Serena Williams or Maria Sharapova use forced, audible exhalation (groaning) for strategical reasons (e.g., to irritate the opponent) and/or to increase force production and hit balls harder (Callison et al. 2014; O'Connell et al. 2014, 2016). Groaning and grunting are believed to enhance trunk stabilization by increasing intra‐abdominal pressure, thereby improving the stiffness of the lumbar vertebrae (Callison et al. 2014). This stabilization can facilitate better force transmission to the upper extremities, resulting in greater force production (Kovacs 2007; Callison et al. 2014; O'Connell et al. 2014, 2016). Furthermore, forced exhalation and the Valsalva maneuver have been shown to enhance isometric muscle strength (Ikeda et al. 2009). Supporting evidence from other sports indicates that grunting is associated with enhanced performance, such as improved deadlift outcomes and increased grip and forearm flexion force in karate (Welch and Tschampl 2012; O'Connell et al. 2014).

Studies on the respiratory patterns in tennis are limited. O'Connell et al. 2014, 2016 demonstrated that forced exhalation and grunting increased isometric force production during forehand strokes. Only the study from Callison et al. (2014) considered the effects of grunting while hitting forehand and backhand strokes on the tennis court and reported an increase in stroke velocity when players grunt. However, there is still no evidence regarding the relation between stroke production, breathing patterns, and oxygen uptake under experimentally controlled conditions. Anecdotal reports from practical settings indicate that some players seem to experience issues with breathlessness during longer rallies, which subsequently affects their performance. Therefore, the aim of this study was to compare respiration patterns and gas exchange during a standardized tennis protocol with increasing hitting and running loads with corresponding measures during continuous treadmill running at a similar mean oxygen uptake.

## 2 Material and Methods

### 2.1 Participants

A total of 15 female and male healthy, competitive tennis players (women *n* = 7: age: 22.6 ± 2.4 years, weight: 69.7 ± 11.9 kg, height: 172.6 ± 8.0 cm, World Tennis Number (WTN): 14.4 ± 2.6; men *n* = 8: age: 25.5 ± 3.1 years, weight: 83.4 ± 8.2 kg, height: 187.4 ± 6.8 cm, WTN: 13.9 ± 3.1) participated in the study. All players held a national ranking within the German Tennis Federation ranking list and, for the purpose of international comparability, were classified according to the ITF WTN (ITF 2025; Martínez‐Gallego et al. 2025). The WTN represents a global and dynamic rating system that quantifies a player's current skill level, independent of age or gender, and allows players to compare their performance levels with competitors worldwide. The WTN ranges from 40 to 1, with beginners starting at 40 and professional players approaching 1. Separate ratings are provided for singles and doubles (ITF 2025; Martínez‐Gallego et al. 2025). Both male and female participants were of a comparable relative performance level. All participants were free of musculoskeletal injuries throughout the study and were familiar with the test procedures and the testing surroundings. Written informed consent was received from all athletes before participation. The study was approved by the ethics committee of the Faculty of Sport Science of the Ruhr University Bochum and in accordance with the declaration of Helsinki (EKS V 2023_13.1).

### 2.2 Study Design and Procedures

**FIGURE 1:**
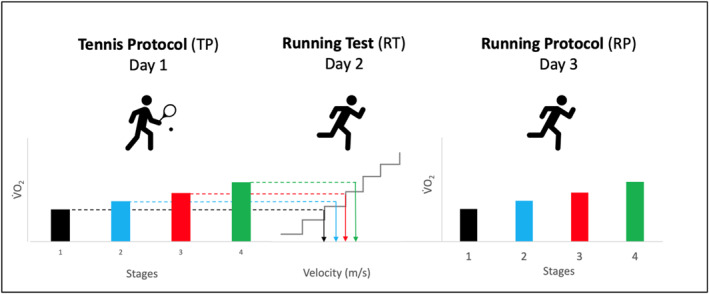
Illustration of the standardized tennis‐court protocol and the procedure for determining individual running speeds for the running protocol (RP, Day 3) under consistent metabolic demand.

On three experimental testing days, on separate occasions, participants completed one standardized ball‐machine‐based hitting tennis protocol (TP, Day 1), a treadmill‐based incremental running test (RT, Day 2), and a standardized running protocol metabolically matched to TP (RP, Day 3, Figure 1). The study took place in an indoor tennis center on a carpet surface and an indoor treadmill in a lab.

**FIGURE 2:**
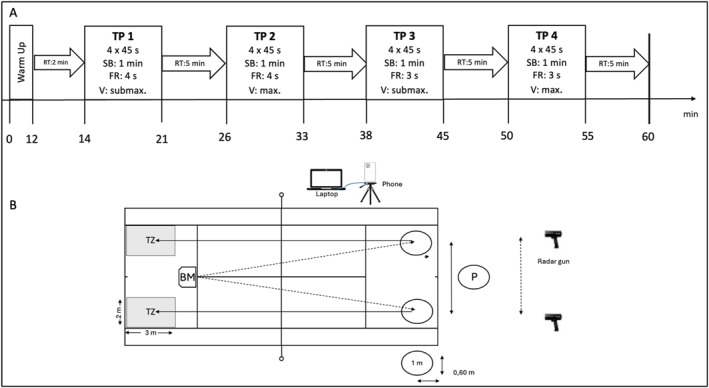
Schematic illustration of the tennis‐court protocol (A, Day 1) displayed as a “number of sets” x “duration of one set” with a timeline and the testing area in the tennis center (B). TP = stage; SB = set break; RT = resting time; FR = frequency of the ball machine; V = velocity of the player's strokes; submax. = submaximal; max. = maximal, P = player; BM = ball‐machine; TZ = target zone.

TP (Day 1) included low or high running loads (RL, RH) and low or high stroke velocities (SL, SH), which were combined in four incremental ball‐machine stages (TP_1_:RL + SL; TP_2_:RL + SH; TP_3_:RH + SL; TP_4_:RH + SH, Figure 2A). The warm‐up included slow forward and backward running, dynamic mobilization exercises, and ended with 5 min hitting groundstrokes. The velocity in the stages was controlled by a ball machine (BM), which was located at the T‐cross. Each stage consisted of four sets and lasted 45 s with 60 s rest between the sets and 5 min break (Figure 2A). The players were instructed to always hit FH and BH strokes longline, down the line in marked target zones (3 × 2 m) (Figure 2B). In TP_1_, players were asked to return balls with low running loads and low stroke velocities (Figure 2A). The frequency of the BM was set at 1 ball every 4 s with a velocity of approximately 60 km/h. All players had the same instruction for this stage: Hit the ball longline into the target zones with alternating forehand (FH) and backhand (BH), as if you would not want to make mistakes under any circumstances in match conditions. For TP_2_, the stroke frequency remained the same; however, the players were required to hit the balls with maximum velocity. The instruction was to go on a longline winner. In TP_3_, hitting frequency was increased to 1 ball every 3 s and the players were instructed to hit the balls with submaximal velocity. In TP_4_, the participants were required to hit the balls maximally while the running load remained the same.

RT (Day 2) was used to determine the running velocity matching the oxygen consumption during each stage of TP (TP_1_‐TP_4_, Figure 1). The incremental treadmill test began individually at a velocity between 2.0 or 3.0 m/s. Treadmill velocity was slightly increased 12 times by 0.2 m/s per every 45 s. For each player mean $\dot{V}O_{2}$ for each stage of TP and each stage of RT were subsequently calculated (Figure 1). These data were used to define the corresponding individual running speeds for the running protocol on Day 3 (RP). RP (Day 3) included four stages with identical length and mean oxygen consumption as during TP.

### 2.3 Measurements

During the three occasions, the participants wore a portable gas measuring system (MetaMax 2B, Cortex, Leipzig, Germany) and a chest belt (H10 Sensor; Polar Electro GmbH Deutschland, Büttelborn, Germany). Breath‐by‐breath analysis was used to determine the volume and composition of each single breath. We calculated the average cardiorespiratory and metabolic parameters (oxygen uptake ($\dot{V}$O_2_), energy expenditure (EE), tidal volume (VT), breathing frequency (bf), minute ventilation ($\dot{V}$’E), inspiration (tI), and expiration time (tE)) as well as stroke velocity (SV) for each 45 s stage of TP and RP. Mean postexercise $\dot{V}$O_2_ was calculated during a 60‐s recovery period after finishing TP and RP. The number (NP) and duration of breathing plateaus (DP) were calculated from VT. Plateaus of VT were defined as “no change in inspiration and expiration for at least 0.2 s by more than + 0.2 l and ‐ 0.2 l”. Moreover, based on NP, two groups of players were classified: plateau player (PP) and nonplateau player (NPP). PP was characterized by an average of three plateaus occurring in at least two protocols and at least one plateau in all protocols. Conversely, players not meeting these criteria were categorized as NPP. During the tennis protocol, stroke velocity was measured using two radar guns (Pro 2 Radar Gun, Stalker, Texas, USA), which were placed 3 m behind and 2 m above the player on the FH or BH side of the court. A ball machine (Spinfire Pro 2, Betzigau, Germany), located at the T‐cross, ensured that every ball was played at the same speed and frequency. A camera (iPhone 12 Pro, Apple, USA), located at the same height as the T‐line outside the court, recorded the players' hitting movements (Figure 2). The phone was connected to the laptop to synchronize the measured breathing patterns with the recorded strokes. Additionally, markers in the gas measuring system were placed at each hitting point.

### 2.4 Statistical Analyses

Statistical analyses were performed using Microsoft Excel (Microsoft Co., Redmond, Washington, USA) and the statistical software jamovi (The Jamovi project, version 2.5.5, Sydney, Australia). Data are presented as mean ± standard deviations (SD) and were tested for normal distribution using the Shapiro–Wilk test. In the case of normal distribution, comparisons were conducted using repeated measures analysis of variance (rANOVA). The rANOVA was used to compare TP and RP in the four stages. Alternatively, the nonparametric Friedman test was used to compare groups. If a significant difference was observed, post hoc tests were carried out using paired *t*‐tests for parametric variables and Wilcoxon tests for nonparametric variables. Differences between plateau and nonplateau players were tested by independent *t*‐tests. Statistical significance was set at *p* < 0.05.

## 3 Results

*External Loads:* The four stages in TP came along with increasing loads corresponding to mean running velocities in RP and corresponded to 3.2 ± 0.2 m/s (TP_1_), 3.5 ± 0.4 m/s (TP_2_), 3.8 ± 0.5 m/s (TP_3_), and 4.2 ± 0.5 m/s (TP_4_). Average stroke velocities ranged between 93 and 122 km/h during submaximal and maximal velocities in FH and BH strokes. Stroke velocities were higher in T_2_ compared to T_1_ (FH: 122.0 ± 8.3 vs. 98.4 ± 8.8; BH: 113.1 ± 7.9 vs. 93.9 ± 7.4 km/h, *p* < 0.001) and in T_4_ compared to T_3_ (FH: 121.5 ± 7.2 vs. 100.1 ± 6.3; BH: 114.5 ± 6.8 vs. 97.8 ± 5.4 km/h, *p* < 0.001).

**FIGURE 3:**
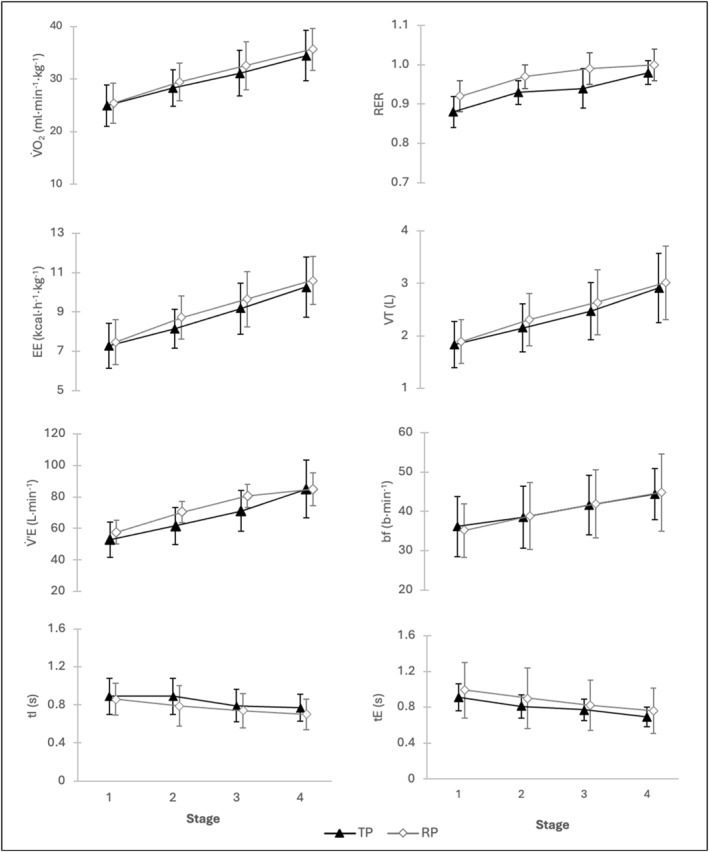
Metabolic and respiratory parameters during a ball‐machine‐based tennis protocol (filled triangles) and a treadmill‐based running protocol (open squares) consisting of four stages of increasing intensity. $\dot{V}$O_2_ = relative oxygen consumption; EE = energy expenditure per hour; RER = respiratory exchange ratio; VT = tidal volume; $\dot{V}$’E = minute ventilation; bf = breathing frequency; tI = inspiration time; tE = expiration time.

*Internal Loads:* The corresponding metabolic and respiratory parameters are presented in Tables 1, and 2. Specifically to the four stages in tennis, significant increases were observed between TP_1_ to TP_4_ in mean $\dot{V}$O_2_ (*p* < 0.001), EE (*p* < 0.001), and RER (*p* < 0.001). No significant differences were found for $\dot{V}$O_2_ (*p* = 0.66), EE (*p* = 0.37), and RER (*p* = 0.34) between the tennis protocol (TP) and the running protocol (RP) (Table 1). Mean RER increased during TP and RP from 0.88 ± 0.0 (TP_1_) and 0.92 ± 0.0 (RP_1_) to 0.98 ± 0.0 (TP_4_) and 1.00 ± 0.0 (RP_4_) (Table 1, Figure 3). Consequently, the estimated relative fat oxidation decreased from an initial value of 36% in TP_1_ and 26% in RP_1_, 26% (TP_2_) and 10% (RP_2_), 16% (TP_3_) and 3% (RP_3_) to 6% in TP_4,_ and almost 0% in RP_4_. Postexercise $\dot{V}$O_2_ was significantly higher in TP (12 from 14 players) compared to RP (TP: 35.5 ± 3.7, RP: 33.2 ± 3.0 ml·min^−1^ kg^−1^, *p* = 0.03). Regarding respiration parameters, no significant differences were observed between TP and RP for VT (*p* = 0.79), bf (*p* = 0.95), $\dot{V}$’E (*p* = 0.43), tI (*p* = 0.79), and tE (*p* = 0.12) (Table 1). The number of plateaus (NP) was significantly higher in TP compared to RP on stages TP_2_ (5.9 ± 3.8 vs. 0.4 ± 0.5 times, *p* < 0.001), TP_3_ (2.6 ± 2.7 vs. 0.3 ± 0.6 times, *p* = 0.04), and TP_4_ (4.6 ± 4.1 vs. 0.3 ± 0.6 times, *p* < 0.001) with no significant differences in TP_1_ (*p* = 0.08). Regarding the stages in TP, an increase was observed from TP_1_ to TP_4_ in VT (1.8 ± 0.4 vs. 2.9 ± 0.7 L/s, *p* < 0.001), bf (36.2 ± 7.7 vs. 44.3 ± 6.6 b·min^−1^, *p* < 0.001), and $\dot{V}$’E (52.9 ± 11.3 vs. 85.0 ± 18.4 L/min, *p* < 0.001), whereas a decrease was found for tI (0.9 ± 0.2 vs. 0.8 ± 0.1 s, *p* < 0.001) and tE (0.9 ± 0.2 vs. 0.7 ± 0.1 s, *p* < 0.001).

**TABLE 1:** Metabolic and respiratory parameters during a ball‐machine‐based tennis protocol (TP) and a treadmill‐based running protocol (RP) consisting of four stages of increasing intensity. rANOVA main effects between TP and RP (protocol) and between the stages are shown.

		Stages				rANOVA Protocol Tennis stages
		1	2	3	4	
$\dot{V}$O_2_ [ml·min^−1^ kg^−1^]	Tennis	25.0 ± 4.0	28.3 ± 3.5	31.1 ± 4.3	34.5 ± 4.8	*p* = 0.66
	Running	25.5 ± 3.8	29.5 ± 3.6	32.5 ± 4.6	35.7 ± 4.0	* *p* < 0.001
EE [kcal·h^−1^·kg^−1^]	Tennis	7.3 ± 1.2	8.2 ± 1.0	9.2 ± 1.3	10.3 ± 1.5	*p* = 0.37
	Running	7.5 ± 1.2	8.7 ± 1.1	9.7 ± 1.4	10.6 ± 1.2	* *p* < 0.001
RER [$\dot{V}$O_2_/$\dot{V}$CO_2_]	Tennis	0.88 ± 0.0	0.93 ± 0.0	0.94 ± 0.1	0.98 ± 0.0	*p* = 0.34
	Running	0.92 ± 0.0	0.97 ± 0.0	0.99 ± 0.0	1.00 ± 0.0	* *p* < 0.001
VT [L]	Tennis	1.8 ± 0.4	2.2 ± 0.5	2.5 ± 0.5	2.9 ± 0.7	*p* = 0.79
	Running	1.9 ± 0.4	2.3 ± 0.5	2.6 ± 0.6	3.0 ± 0.7	* *p* < 0.001
NP	Tennis	3.1 ± 3.0	5.9 ± 3.8	2.6 ± 2.7	4.9 ± 4.1	* *p* < 0.001
	Running	0.6 ± 0.8	0.4 ± 0.5	0.3 ± 0.6	0.3 ± 0.6	* *p* < 0.001
DP [s]	Tennis	0.48 ± 0.18	0.54 ± 0.18	0.49 ± 0.25	0.50 ± 0.18	*p* = 0.78
$\dot{V}$'E [L·min^−1^]	Tennis	52.9 ± 11.3	61.6 ± 11.8	71.1 ± 13.1	85.0 ± 18.4	*p* = 0.43
	Running	57.6 ± 7.4	70.4 ± 6.7	80.7 ± 7.2	93.7 ± 10.4	* *p* < 0.001
bf [breaths · min^−1^]	Tennis	36.2 ± 7.7	38.6 ± 7.9	41.6 ± 7.6	44.3 ± 6.6	*p* = 0.95
	Running	35.2 ± 6.1	38.8 ± 8.2	42.0 ± 8.6	44.8 ± 9.8	* *p* < 0.001
tI [s]	Tennis	0.89 ± 0.19	0.89 ± 0.19	0.79 ± 0.17	0.77 ± 0.14	*p* = 0.79
	Running	0.86 ± 0.17	0.79 ± 0.21	0.74 ± 0.18	0.70 ± 0.16	* *p* < 0.001
tE [s]	Tennis	0.91 ± 0.15	0.81 ± 0.13	0.77 ± 0.12	0.69 ± 0.11	*p* = 0.12
	Running	0.99 ± 0.31	0.90 ± 0.34	0.82 ± 0.28	0.76 ± 0.25	* *p* < 0.001

*Note:* Values described as average ± standard deviation.

Abbreviations$:\dot{V}$’E = minute ventilation; $\dot{V}$O_2_ = relative oxygen consumption; bf = breathing frequency; EE = energy expenditure per hour; RER = respiratory exchange ratio; tE = expiration time; tI = inspiration time; VT = tidal volume.

^*^ significant difference between the tennis and running protocol and the tennis stages.

**TABLE 2:** Metabolic and respiratory parameters as well as external loads in male and female players during a ball‐machine‐based tennis protocol (TP) and a treadmill‐based running protocol (RP) consisting of four stages of increasing intensity. rANOVA main effects of gender are shown.

		Stages								rANOVA *Gender
		1		2		3		4		
		m	w	m	w	m	w	m	w	
$\dot{V}$O_2_ [ml·min^−1^ kg^−1^]	Tennis	25.7 ± 3.9	24.1 ± 4.1	29.1 ± 3.5	27.4 ± 3.5	32.0 ± 4.3	30.1 ± 4.4	36.0 ± 5.5	32.7 ± 3.5	* *p* = 0.03
	Running	26.8 ± 3.0	23.9 ± 4.3	30.4 ± 3.3	28.4 ± 3.8	33.6 ± 4.5	31.4 ± 4.8	37.1 ± 3.6	34.0 ± 4.1	
EE [kcal·h^−1^ kg^−1^]	Tennis	7.5 ± 1.1	7.0 ± 1.2	8.2 ± 1.0	8.0 ± 1.0	9.4 ± 1.3	8.9 ± 1.3	10.8 ± 1.8	9.7 ± 1.1	* *p* < 0.001
	Running	7.9 ± 0.9	7.0 ± 1.3	9.0 ± 1.0	8.4 ± 1.1	9.9 ± 1.4	9.3 ± 1.4	11.0 ± 1.1	10.1 ± 1.2	
RER [$\dot{V}$O_2_/$\dot{V}$CO_2_]	Tennis	0.88 ± 0.0	0.89 ± 0.1	0.92 ± 0.0	0.93 ± 0.0	0.93 ± 0.1	0.95 ± 0.0	0.98 ± 0.0	0.99 ± 0.0	*p* = 0.11
	Running	0.92 ± 0.1	0.91 ± 0.0	0.96 ± 0.0	0.98 ± 0.0	0.98 ± 0.1	1.00 ± 0.0	0.99 ± 0.1	1.02 ± 0.0	
VT [L]	Tennis	2.0 ± 0.4	1.6 ± 0.4	2.3 ± 0.4	1.9 ± 0.4	2.7 ± 0.6	2.2 ± 0.4	3.2 ± 0.7	2.5 ± 0.4	* *p* < 0.001
	Running	2.0 ± 0.4	1.6 ± 0.3	2.4 ± 0.5	2.1 ± 0.5	2.8 ± 0.7	2.4 ± 0.6	3.1 ± 0.8	2.7 ± 0.6	
NP	Tennis	2.8 ± 3.4	3.2 ± 3.1	5.2 ± 4.4	6.3 ± 3.6	2.9 ± 3.2	2.2 ± 1.8	4.1 ± 3.5	5.6 ± 4.7	*p* = 0.87
	Running	0.7 ± 0.9	0.4 ± 0.6	0.3 ± 0.6	0.2 ± 0.3	0.4 ± 0.7	0.2 ± 0.4	0.3 ± 0.6	0.3 ± 0.5	
DP [s]	Tennis	0.49 ± 0.18	0.45 ± 0.16	0.58 ± 0.21	0.50 ± 0.10	0.48 ± 0.35	0.48 ± 0.14	0.57 ± 0.19	0.44 ± 0.13	*p* = 0.34
$\dot{V}$'E [L·min^−1^]	Tennis	57.4 ± 8.9	47.8 ± 12.1	66.6 ± 7.3	55.9 ± 13.9	77.0 ± 11.0	64.4 ± 12.6	93.8 ± 17.0	75.0 ± 15.3	* *p* < 0.001
	Running	64.3 ± 10.0	49.9 ± 99.6	76.1 ± 12.2	63.9 ± 15.0	87.5 ± 17.5	72.9 ± 17.0	99.8 ± 18.4	84.1 ± 15.8	
bf [breaths · min^−1^]	Tennis	34.6 ± 6.0	38.7 ± 9.5	36.6 ± 7.9	41.1 ± 8.5	39.2 ± 6.7	45.1 ± 8.5	41.5 ± 5.2	47.1 ± 7.3	* *p* < 0.001
	Running	33.0 ± 6.0	38.6 ± 5.4	35.5 ± 7.1	43.7 ± 6.5	39.0 ± 8.4	46.7 ± 7.7	40.0 ± 8.6	51.4 ± 8.7	
tI [s]	Tennis	0.95 ± 0.17	0.84 ± 0.21	0.94 ± 0.20	0.84 ± 0.17	0.86 ± 0.17	0.71 ± 0.14	0.79 ± 0.18	0.74 ± 0.10	* *p* < 0.001
	Running	0.92 ± 0.19	0.79 ± 0.12	0.87 ± 0.22	0.70 ± 0.11	0.80 ± 0.20	0.67 ± 0.14	0.79 ± 0.14	0.61 ± 0.12	
tE [s]	Tennis	0.96 ± 0.14	0.84 ± 0.15	0.87 ± 0.12	0.75 ± 0.11	0.83 ± 0.11	0.71 ± 0.09	0.76 ± 0.09	0.62 ± 0.08	* *p* < 0.001
	Running	1.09 ± 0.40	0.88 ± 0.12	0.99 ± 0.41	0.78 ± 0.11	0.91 ± 0.37	0.73 ± 0.11	0.87 ± 0.30	0.64 ± 0.08	
SV [km/h]	FH	99.2 ± 9.7	97.6 ± 8.4	126.3 ± 8.2	117.2 ± 5.6	99.9 ± 6.8	100.3 ± 6.2	123.8 ± 7.9	119.0 ± 5.9	*p* = 0.29
	BH	95.4 ± 8.2	92.2 ± 6.6	116.9 ± 7.9	108.7 ± 5.6	98.5 ± 5.0	97.0 ± 6.1	117.5 ± 5.8	111.2 ± 6.5	*p* = 0.10

*Note:* Values described as average ± standard deviation.

Abbreviations: $\dot{V}$’E = minute ventilation; $\dot{V}$O2 = relative oxygen consumption; bf = breathing frequency; BH = backhand; EE = energy expenditure per hour; FH = forehand; RER = respiratory exchange ratio; SV = Stroke velocity; tE = expiration time; tI = inspiration time; VT = tidal volume.

^*^ significant difference between male and female players.

*Gender Effects:* Gender‐related comparisons revealed that male players reached a higher average $\dot{V}$O_2_ (*p* = 0.03), EE (*p* < 0.001), VT (*p* < 0.001), $\dot{V}$’E (*p* < 0.001), tI (*p* < 0.001), and tE (*p* < 0.001), whereas female players had higher bf (*p* < 0.001), and RER remained similar (Table 2). The number (NP), duration of plateaus (DP) and SV FH and BH did not differ between genders (Table 2).

**FIGURE 4:**
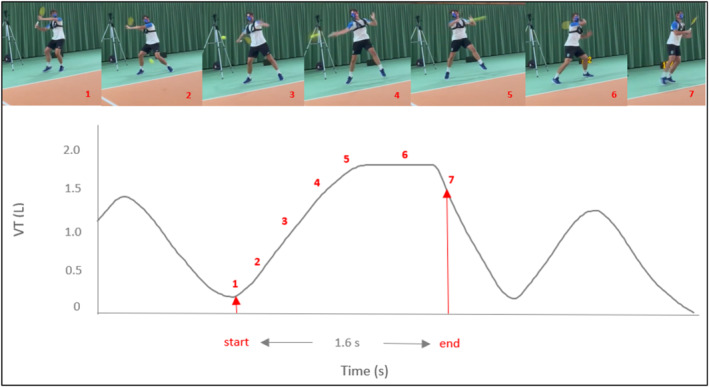
Image series (1–7) of a FH stroke (top) with the synchronized breathing curve (bottom).

**FIGURE 5:**
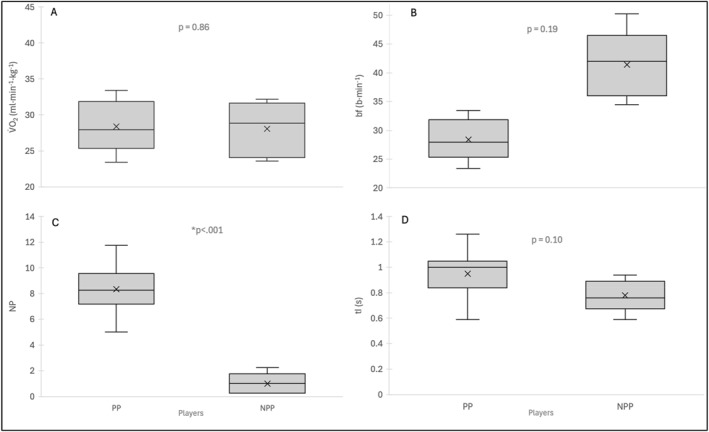
Difference between metabolic and respiratory parameters and external factors in Stage 2 of the tennis protocol between plateau players (PP, *n* = 10) and nonplateau players (NPP, *n* = 5). $\dot{V}O_{2}$ = relative oxygen uptake; bf = breathing frequency; NP = number of plateaus; tI = inspiration time.

*Breathing Plateaus:* Plateaus occurred on average 3.1 ± 3.0 times (TP_1_), 5.9 ± 3.8 times (TP_2_), 2.6 ± 2.7 times (TP_3_), and 4.9 ± 4.1 times (TP_4_) with an average duration ranging around 0.5 s (Table 1). These plateaus tended to happen around the hitting point until the end after completing the stroke while pushing back to the center of the court (Figure 4, picture 6). During stroke preparation and until the hitting point, players were observed to inhale (Figure 4, pictures 1–4). NP was higher in stages with high stroke velocities (TP_2 and 4_) compared to stages of slow stroke velocities (TP_1 and 3_, *p* < 0.001) (Table 1). The duration of plateaus (DP) did not differ between the stages (*p* = 0.78). Ten players (5 women, 5 men) could be classified as plateau players (PP), whereas five players (2 women, 3 men) were categorized as nonplateau players (NPP). PP did have higher NP than NPP (8.35 ± 1.78 vs. 1.00 ± 0.74 times, *p* < 0.001), whereas no difference was found for oxygen consumption, post‐oxygen consumption (PP: 35.3 ± 2.2 vs. NPP: 35.8 ± 5.6 ml·min^−1^ kg^−1^, *p* = 0.68) and stroke velocity. NPP tended to have a higher breathing frequency and a shorter inspiration time (Figure 5).

## 4 Discussion

The present study provides a first insight in the respiration patterns during a standardized tennis protocol with corresponding measures during a metabolically matched treadmill running protocol. The main difference between tennis and running is that two‐thirds of tennis players hold their breath for about half a second around the hitting point especially at higher stroke velocities. Interestingly, this belongs likewise to males and females and had no impact on oxygen uptake and stroke velocity between groups.

The results in the present study revealed a high internal load (e.g., $\dot{V}$O_2_, EE, and RER) during the 4 times 7 min workloads with no significant differences between tennis and running. From a methodological perspective, this indicates that the metabolic alignment between the two protocols was successful, fulfilling the basic requirement for a valid comparison between tennis and running. Compared to other studies, the intensity of the tennis protocol was clearly higher. During a 2‐h tennis match play, lower average $\dot{V}$O_2_ values (25.6 ml·min^−1^ kg^−1^) were reported (Ferrauti et al. 2001). The differences can be attributed to the special structure of tennis match play consisting of unpredictable load peaks, short rallies in average (3–8 s) followed by longer recovery periods, especially during the change of ends (Fernandez‐Fernandez et al. 2010; Edel et al. 2019). In the present study, the rest periods between the stages were not included in the calculation. Consequently, we calculated higher running speeds on treadmill as the corresponding running load to the tennis protocol compared to previous studies (Ferrauti et al. 2001). Since our aim was to imitate longer rallies and intensive phases during match play (45 s), rather than the average match play load, we assume that our protocol is suitable to investigate breathing patterns in tennis in extremely demanding situations.

**FIGURE 6:**
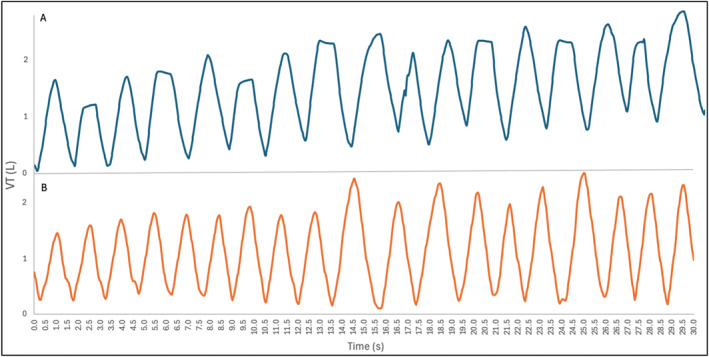
Excerpt from a 30 s breathing pattern in TP_2_ with high stroke velocities (A) and a breathing pattern in RP_2_ (B).

With increasing exercise intensity, breathing patterns in both protocols changed with an almost linear increase in V’E, VT, and bf and a decrease in inspiration and expiration time (Figure 6). Interestingly, no differences were found for the respiration parameters between TP and RP. In line with that, Naranjo et al. (2005) analyzed respiratory parameters between a ramp and a step protocol. The study suggests a consistent response in ventilation regardless of whether a ramp or step protocol is used. The authors argue that, irrespective of the participants' training level or differences in protocols, ventilation behaves similarly after a central order (activation of VT and tI) at a given intensity effort (Naranjo et al. 2005). Applied to the current study, this could be one explanation why the respiratory parameters generally do not differ between TP and RP.

During the four stages in tennis, there were significant changes in all respiratory and metabolic parameters from TP_1_ to TP_4_, suggesting that both an increase in running velocity as well as higher stroke velocities seem to have an impact on energetic and respiratory demands. In accordance with this, Fernandez‐Fernandez et al. (2010) showed that high groundstroke velocities could increase oxygen consumption up to 85% of $\dot{V}$O_2max_. This could be due to the engagement of upper body muscles necessary for executing the ball stroke, as well as the activation of leg muscles (e.g., biceps femoris, rectus femoris, hip adductors), which are highly active during the stroke position (Fernandez‐Fernandez et al. 2010; Edel et al. 2019).

The main finding of this study was that breathing plateaus occurred exclusively in the tennis protocol, where the inhalation and exhalation phases were interrupted by a plateau of approximately 0.5 s around the hitting point (Table 1). As expected, no breathing plateaus occurred during the running protocol. Instead, there is a rhythmic alternation between inhalation and exhalation probably coordinated by the step cadence (Harbour et al. 2023; van Rheden et al. 2023). Interestingly, the number of plateaus was higher in stages with high stroke velocities (TP_2_ and TP_4_) compared to slow stroke velocities (TP_1_ and TP_3_). This suggests that the plateaus come along with press breathing when aiming to achieve high stroke velocities. Press breathing, also known as the Valsalva maneuver, is a breathing technique involving exhaling against a closed glottis, thereby increasing the pressure in the chest and abdomen (Davies et al. 2020). This technique is believed to enhance athletic performance by improving trunk stability, consequently enhancing the ability to generate and transfer force from larger to smaller body parts (Callison et al. 2014). The proposed mechanism involves an elevation in intra‐abdominal pressure during respiration, leading to an increased stiffness of the lumbar vertebrae and enhanced trunk stability, ultimately contributing to greater force development (Ikeda et al. 2009; Callison et al. 2014).

Two thirds of the players could clearly be identified as plateau players (Figure 6). This shows that most of the players, but not all players, are taking advantage of the described mechanisms and that players apparently find individually different solutions to an identical task. Unfortunately, we were unable to distinguish between other breathing variants, such as forceful exhalation or grunting. The latter phenomenon likely occurs and may also enhance core stiffness, which in turn can influence stroke velocity. This is particularly relevant in light of findings by Callison et al. (2014), which investigated whether grunting increased ball velocity by instructing players to grunt during one hitting session and refrain from grunting in another. The results revealed that players who grunt had higher ball velocities. Moreover, nongrunters achieved higher stroke velocities in their groundstrokes with minimal practice of grunting. Grunting in the sense of constricting the glottis without completely interrupting breathing can therefore also be a suitable alternative. However, no acoustic data were collected in the present study, limiting the ability to differentiate between these mechanisms. This uncertainty may also help explain why plateau players did not necessarily achieve higher stroke velocities compared to nonplateau players. Furthermore, it must be considered that high hitting velocities are primarily depending on the individual technical skills and the small sample size of only 10 plateau and 5 nonplateau players may have further obscured potential differences between groups. Future research should incorporate synchronized respiratory and acoustic recordings to clarify the distinct contributions of breath holding and grunting to stroke velocity.

Surprisingly, the study revealed that PP did not differ in $\dot{V}$O_2_ and postexercise $\dot{V}$O_2_ compared to NPP. This contradicts the previous suggestion that a higher number of plateaus in the tennis protocol may result in higher postexercise $\dot{V}$O_2_ values. Moreover, the duration of the plateaus between the stages did not differ. We assume, that players who regular perform press breathing learn to cope with the partially reduced oxygen availability during their carrier by compensating for it between the plateau phases.

Nevertheless, notable differences were found for the postexercise oxygen consumption between the tennis and running protocols showing significantly higher values in tennis. This might be explained by high‐intensive work‐load peaks occurring during the more intensive stages in tennis partly demanding anaerobic energy supply coming along with an accumulated oxygen deficit. Moreover, the intermittent exercise profile in tennis with the involvement of stroke activities differs compared to continuous exercises like running and could also have an impact on postexercise oxygen demand. On the other hand, it cannot be ruled out that the breathing plateaus in tennis are partly responsible. It has been shown that they come along with an increased activation of trunk stabilizers and higher abdominal pressure against the diaphragm and induced an increased activation of mechanoreceptors within the muscle (Reinhard et al. 2020). This could result in a brief oxygen deficit and therefore an increased postexercise oxygen demand.

In female players, lower values for $\dot{V}$O_2_, EE, VT, $\dot{V}$’E, tI, and tE were observed (Table 2). This may be the result of lower energy requirements, which, among other things, is associated with forehand and backhand strokes that are 5–10 km/h slower. However, breathing frequency was higher in females. This could be explained by smaller airways and lung volume compared to men which may lead to an increased bf (Dominelli et al. 2018; Reinhard et al. 2020). The number of PP (5 women and 5 men) and NPP (2 women and 3 men) was equally distributed in both sexes. Obviously, players, regardless of gender, make use of increased trunk stability to enhance force production (Callison et al. 2014).

### 4.1 Limitations

A central limitation of the study is that the exact alignment of the plateau with the hitting point remains uncertain due to limited temporal synchronization between breath analysis and video analysis. As shown in Figure 4, breathing plateaus occasionally appeared to last into the player's movement back to the center of the court. This temporal inaccuracy may partly be explained by latency between the mobile spirometry device and the laptop caused by the Bluetooth connection, which could have led to a systematic time delay. This methodological uncertainty does not negate the consistent occurrence of plateaus in the tennis protocol, but it limits the strength of the conclusion that they are directly linked to ball contact. Therefore, strokes could trigger characteristic breathing interruptions, yet further studies with more precise synchronization methods are required to confirm their exact timing in relation to strokes. Observations during official tournament matches of players who vocalize their exhalation suggest that forced breathing is often released only after the impact point.

A second limitation was that the players were not tested in a cross‐over design using both press breathing and non‐press breathing, which would have allowed a clearer evaluation of the effect on stroke velocity. Finally, RPE values were not included in the study. Therefore, no statements could be made regarding the subjective perception of exertion. Further studies should consider these limitations.

### 4.2 Practical Applications

Previous studies have already shown that the exclusive aerobic running exercise for tennis players is not suitable due to the different internal and external loads, including the body's entire muscular system and the involvement of stroke mechanism in tennis (Ferrauti et al. 2001; Fernandez‐Fernandez et al. 2010). Additionally, respiratory patterns seem to differ between tennis and running and should also be considered and improved during practice. During on‐court drills, it is recommended to explore individual effects of press‐breathing and/or grunting in combination with running activities. Plateau players should practice this technique and get used to the short‐term oxygen deficits.

## 5 Conclusion

Respiration patterns differ significantly between tennis and running despite a similar mean oxygen consumption. Breathing plateaus occur exclusively in tennis and are used by most players without increasing the postexercise oxygen consumption. This indicates that plateau players most likely compensate short‐term oxygen deficits between the strokes. Synchronization between respiratory data and video analysis showed that breathing plateaus occur during a time window around the hitting point. For players and coaches in tennis, it could be beneficial to explore different breathing patterns during on‐court drills as a means of individual performance enhancement.

## Author Contributions

K.R., A.E., and A.F. have made substantial contributions to conception and design. K.R. was responsible for acquisition of data, data analysis and interpretation of data. A.E. and A.F. were involved in interpretation of the data. All authors have been involved in drafting the manuscript or revising it critically. All authors participated sufficiently in the work, have given final approval of the version to be published, take public responsibility for appropriate portions of the content, and agreed to be accountable for all aspects of the work in ensuring that questions related to the accuracy or integrity of any part of the work are appropriately investigated and resolved.

## Funding

This study received no external funding.

## Ethics Statement

The study design, procedures, and measurements aligned with the Declaration of Helsinki and were approved by the local ethics committee of the Faculty of Sports Science at Ruhr University Bochum (EKS V 2023_13.1).

## Consent

All participants were informed about the testing procedures, data policy, and potential risks of the study and gave their written informed consent to voluntarily participate.

## Conflicts of Interest

The authors declare no conflicts of interest.

## Permission to Reproduce Material From Other Sources

The article contains no material that has been reproduced from any other sources.

## Data Availability

The data that support the findings of this study are available from the corresponding author upon reasonable request.
